# VUS next in rare diseases? Deciphering genetic determinants of biomolecular condensation

**DOI:** 10.1186/s13023-024-03307-6

**Published:** 2024-09-06

**Authors:** María Heredia-Torrejón, Raúl Montañez, Antonio González-Meneses, Atilano Carcavilla, Miguel A. Medina, Alfonso M. Lechuga-Sancho

**Affiliations:** 1 Inflammation, Nutrition, Metabolism and Oxidative Stress Research Laboratory, Biomedical Research and Innovation Institute of Cadiz (INiBICA), Cadiz, Spain; 2 Department of Molecular Biology and Biochemistry, University of Malaga, Andalucía Tech, E-29071 Málaga, Spain; 3 Division of Dysmorphology, Department of Paediatrics, Virgen del Rocio University Hospital, Sevilla, Spain; 4 Department of Paediatrics, Medical School, University of Sevilla, Sevilla, Spain; 5 Pediatric Endocrinology Department, Hospital Universitario La Paz, 28046 Madrid, Spain; 6 Multidisciplinary Unit for RASopathies, Hospital Universitario La Paz, 28046 Madrid, Spain; 7 Biomedical Research Institute and nanomedicine platform of Málaga IBIMA-BIONAND, E-29071 Málaga, Spain; 8 CIBER de Enfermedades Raras (CIBERER), Instituto de Salud Carlos III, E-28029 Madrid, Spain; 9 Division of Endocrinology, Department of Paediatrics, Puerta del Mar University Hospital, Cádiz, Spain; 10 Area of Paediatrics, Department of Child and Mother Health and Radiology, Medical School, University of Cadiz, Cadiz, Spain; 11 Mother and Child Health and Radiology Department. Area of Clinical Genetics, University of Cadiz. Faculty of Medicine, Cadiz, Spain

**Keywords:** Biomolecular condensation, Genetic variant prioritization, Intrinsically disordered protein regions, LLPS, Molecular diagnosis, Molecular effects of genetic variations, Rare diseases

## Abstract

The diagnostic odysseys for rare disease patients are getting shorter as next-generation sequencing becomes more widespread. However, the complex genetic diversity and factors influencing expressivity continue to challenge accurate diagnosis, leaving more than 50% of genetic variants categorized as variants of uncertain significance.

Genomic expression intricately hinges on localized interactions among its products. Conventional variant prioritization, biased towards known disease genes and the structure-function paradigm, overlooks the potential impact of variants shaping the composition, location, size, and properties of biomolecular condensates, genuine membraneless organelles swiftly sensing and responding to environmental changes, and modulating expressivity.

To address this complexity, we propose to focus on the nexus of genetic variants within biomolecular condensates determinants. Scrutinizing variant effects in these membraneless organelles could refine prioritization, enhance diagnostics, and unveil the molecular underpinnings of rare diseases. Integrating comprehensive genome sequencing, transcriptomics, and computational models can unravel variant pathogenicity and disease mechanisms, enabling precision medicine. This paper presents the rationale driving our proposal and describes a protocol to implement this approach. By fusing state-of-the-art knowledge and methodologies into the clinical practice, we aim to redefine rare diseases diagnosis, leveraging the power of scientific advancement for more informed medical decisions.

**Supplementary Information:**

The online version contains supplementary material available at 10.1186/s13023-024-03307-6.

## The idea

The diagnosis of rare diseases (RDs) remains a challenging and complex endeavor. The genetic diversity present among the nearly 8 billion living humans, with 5·10^6^ variants on average [1], hinders the understanding of genetic traits [2, 3]. This is reinforced by the intricate genetic regulation and the complex interplay of factors that modulate expressivity in RDs. Therefore, it is unsurprising that more than half of genetic variants are considered variants of uncertain significance (VUS) [4, 5], with patients of non-European descendants bearing the brunt [6, 7]. In RDs, this abundance of VUSs is especially significant because it is estimated that around 80% of them have a genetic basis [8].

In the last two decades, genetics has made significant progress, revealing new gene-disease associations, causative molecular mechanisms, and therapeutic developments [9]. However, the intricate interplay of extensive genetic diversity, variable expressivity, and incomplete penetrance in RDs hampers genetic diagnosis and the establishment of clinical relevance in variants related to conditions. A task that is further complicated by the influence of genetic background diversity, epigenetic modifications, environmental factors, and the limited number of cases [10]. Even identical causal variants over different genetic backgrounds can lead to diverse pathological phenotypes, This complexity requires physicians to apply “clinical diagnostic criteria” a set of phenotypic characteristics that patients with a given genetic variant may exhibit in different numbers and degrees. For example, a patient with a pathogenic variant in *PTPN11* (MIM #176876) causing Noonan syndrome may have pulmonary stenosis, while a different patient with the same variant may have a different congenital heart disease, or even no heart disease but shows many other Noonan syndrome characteristics [11]. Conversely, some individuals with disease-causing alleles remain healthy despite affected family members in the same environment [10, 12]. Moreover, the lack of comprehensive studies and adequate tools to gather and analyze this information hinders our ability to fully understand the pathological significance of genetic variants. As a result, diagnosing RDs remains a relevant challenge despite advances in genetic medicine [13].

Identifying the causative variant and mode of inheritance is mandatory to guide a patient’s clinical management, inform patients about related risks, and aid in evaluating family planning options. Unfortunately, diagnosis is long delayed, depending on the patient’s phenotype, age, and resources. On average, it takes around 4–5 years to accurately diagnose a specific RD, but in some cases, a definitive diagnosis can take more than a decade or even die without it [14–17]. Patients often undergo costly and extensive evaluations at multiple institutions and may remain undiagnosed or misdiagnosed, causing emotional distress to patients and relatives. Fortunately, as our understanding of mechanisms behind phenotypic causation advances, new pieces of the puzzle emerge, and we will become better equipped to generate and experimentally verify hypotheses regarding the origins of this phenotypic variability.

The reductionist approach in prioritizing variants, focusing only on well-known disease-causing genes, hinders genetic diagnosis. Despite Fisher’s seminal work proposing polygenic inheritance in 1918 [18] and later validated [19, 20], most variant prioritization algorithms persist in a gene-to-gene approach. But, navigating the complex pathways connecting genotypes to phenotypes requires more comprehensive approaches to avoid uncertain significance scenarios. Genome-wide association studies have further supported the necessity of a systemic view by demonstrating that common SNPs contribute to the genetic architecture of multifactorial traits [21–23]. These variants may affect genes not directly linked to a specific disease, but their cumulative effect may ultimately impact the resulting phenotype [24]. Our adherence could be due to our limited comprehension of the emergent properties arising from epistatic interactions, as well as the need to facilitate clinical management.

Although we are starting to analyze epistatic cross-regulation mediated by nonadditive gene-to-gene interactions, this remains largely unexplored in the prioritization of genetic variants. The individual actions of each of our genes are limited, but collective behavior arises as a result of their local interactions, giving rise to a complex organization [25–27]. Thereby, we must consider the genome as a whole, without overlooking that it comprises individual pieces that coordinate this collective behavior. This complexity may hinder the precise elucidation of the exact number of genes involved and their contributions to phenotypes. To comprehensively understand complex traits, alternative approaches beyond current genotypic analysis are needed. Therefore, we propose an open-minded approach, exploring innovative strategies and thoroughly investigating all variants impacting specific molecular self-organization.

**Fig. 1:**
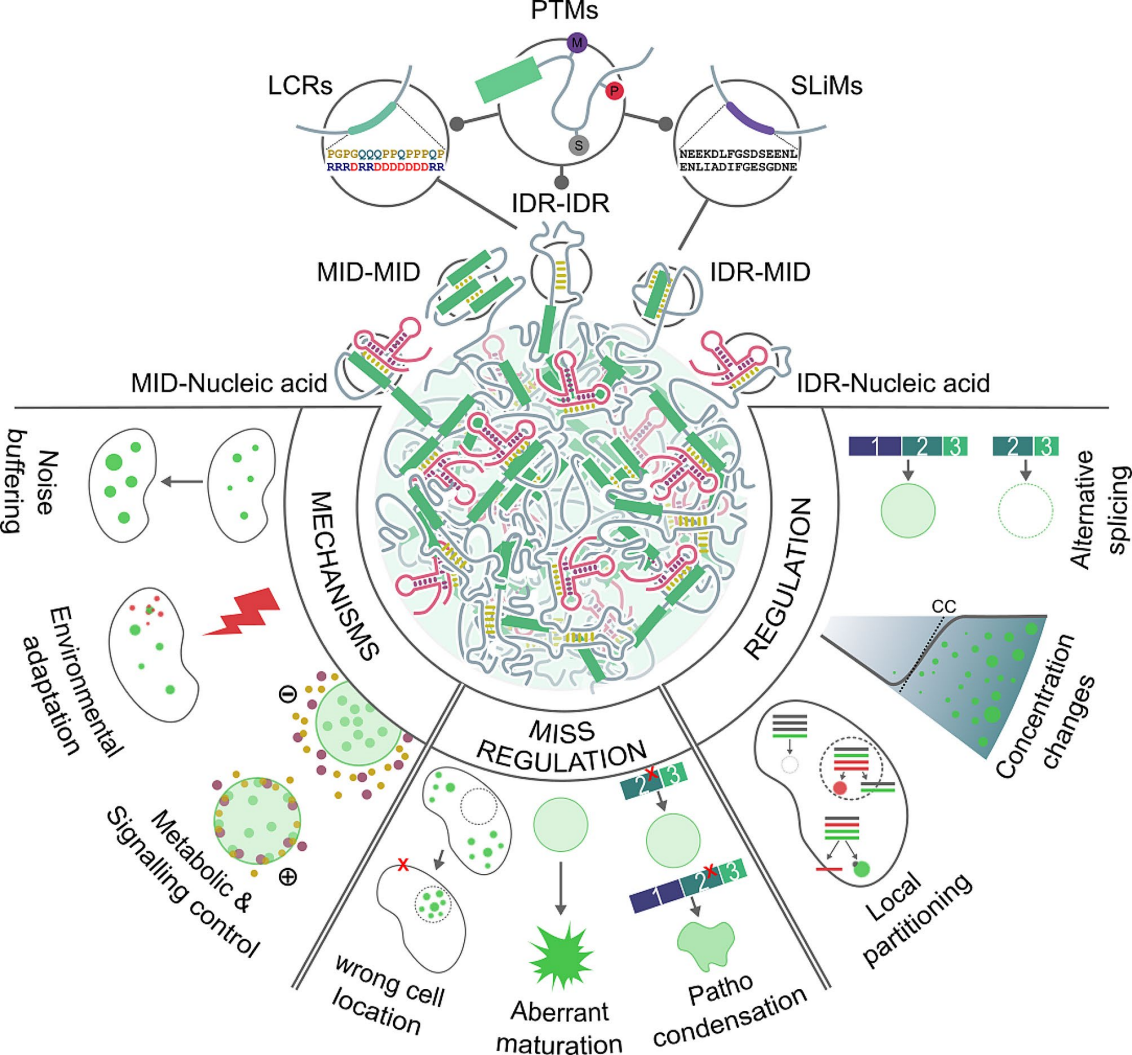
Schematic representation of the factors that modulate the LLPS dynamics in the formation of biomolecular condensates and the potential consequences of variants affecting IDRs. **LCRs**: The low-complexity regions exhibit a limited range of amino acid compositions, leading to reduced amino acid diversity within these regions. Locally, amino acids tend to cluster, forming hydrophobic or electrostatic patches that facilitate the molecular aggregation process; **SLiMs**: short linear interacting motives, **PTMs**: Post-translational modifications, **MID**: modular interacting domain, **IDR**: Intrinsically disordered regions, **CC**: critical concentration

Analyzing patient variants requires considering gene products beyond transcriptional regulation or catalytic activities. Proteins, RNAs, or their combinations operate in crowded environments with competitive molecular interactions. Understanding the collective behavior of genetic diseases relies on two key elements: intrinsically disordered regions (IDRs) [28] and biomolecular condensates (BCs) [29]. Both IDRs and BCs have emerged as significant contenders in unraveling the mysteries of conditions such as cancer, neurodegenerative disorders, or RDs [30–35]. The dysregulation of IDRs and BCs presents an intriguing enigma (see Fig. 1) that requires further exploration. However, their integration into routine clinical practice remains unexplored despite their potential impact. Beyond diagnostic and therapeutic applications, IDRs and BCs offer valuable insights into complex phenomena like variable expressivity and epistasis, which are characteristic of RDs.

## Why explore the effects of variants in IDRs?

In the 1960s, the experiments of biochemist Christian B. Anfinsen established the sequence-structure-function paradigm [36]. The sequence-structure-function paradigm proposes that a protein’s primary amino acid sequence dictates its three-dimensional structure and function. However, biology has shown exceptions, such as the IDRs or intrinsically disordered proteins (IDPs) that lack a fixed three-dimensional structure and exhibit a wide range of conformations and functions [28, 37].

Unlike the conventional protein structure-function model, half of the proteome still performs cellular functions without fully or partially well-defined three-dimensional structures under physiological conditions [38, 39]. In humans, fully folded proteins (37%) or IDPs (5%) only represent the two ends of the continuum [40]. Most human proteins (58%) contain folded protein domains and IDRs [41]. But, so far, little attention has been paid to IDRs or IDPs [37]. However, IDRs deviate from the classical paradigm and have led to the emergence of the disorder-function paradigm that postulates that proteins can remain unfolded while still carrying out their essential physiological functions [34].

IDRs exhibit a captivating functional diversity, spanning up to 8 distinct subtypes [42]. Of particular interest is the fact that these functional subtypes may individually manifest or coexist within a single protein, showing the intricate complexity that can arise from the convergence of multiple disordered regions. This interplay among IDRs highlights their pivotal role in shaping the multifaceted functionality of proteins.

Additional evidence suggests that we should pay more attention to IDRs. Computational analyses estimate that approximately 14% of the proteome in archaea and bacteria, and a substantial portion ranging from 44 to 54% in eukaryotes, consists of disordered regions [43]. Moreover, evolutionary trends reveal that as the genome’s complexity increases, so does the proportion of IDRs within the proteome, particularly during the transition from prokaryotic to eukaryotic life [44, 45]. Furthermore, IDRs tend to be enriched in proteins performing complex functions like signaling, while they are depleted in proteins with more structure-dependent functions, such as metabolic proteins [39, 46]. The fundamental attribute of these sequences is their capacity to regulate and modify protein activity, enabling adaptive responses to diverse situations. This is achieved thanks to the conformational heterogeneity of facilitating proteins, which influences their interactions with other molecules [34].

Notably, IDRs are estimated to be involved in over 20% of genetic diseases on average but can be increased to 50%, such as in skeletal disorders [40]. Focusing on disordered charged biased proteins, 95% of them are associated with multiple diseases [47]. Furthermore, up to 25% of documented disease mutations have been identified within IDRs [48]. Mutations in IDPs such as β-amyloid, α-synuclein, and FUS, have emerged as key contributors to a spectrum of neurodegenerative diseases [49]. These alterations disrupt the interaction dynamics of these IDPs, prompting their aberrant aggregation and, consequently, instigating the pathogenesis of disorders such as Alzheimer’s Disease [49], Parkinson’s Disease [50], or amyotrophic lateral sclerosis [51].

Moreover, the adaptive and regulatory capacity of IDRs is further supported by observed facts related to alternative splicing events [34, 52–54]. Proteins containing tissue-specific exons exhibit a higher average number of interaction partners and serve as central hubs in protein-protein interaction networks [34, 53]. Furthermore, these differentially expressed exons are enriched in IDRs [53]. In addition, IDRs exhibit conserved linear interactive motifs and post-translational modification sites. Hence, tissue-specific splicing of exons facilitates the rewiring of protein-protein interaction networks, enabling adaptation to environmental cues and changing the nature of the response itself. IDRs evolve faster than structured segments due to the reduced constraints on amino acid substitution [55–58], resulting in a higher frequency of variants in these regions.

Structured domains and IDRs should be considered as two functional components of proteins [42]. However, variants within IDRs are often underestimated, leading to their frequent classification as VUS, as a result of the structure-function paradigm use. This limitation hinders our understanding of protein functionality and its implications for human health. The up to now presented facts leave unanswered questions about the functional consequences of variants impacting IDRs in disease contexts and how those IDRs execute specific functions without well-defined structures. Variant-interpretation criteria are applied regardless of whether the region is structured or disordered. Studies carried out in the field of cancer and evolution, point out that folded domains and IDRs differ in terms of their tolerance to mutations [58]. IDRs can display higher tolerance to sequence variations, as they don’t rely on a specific structure to function. However, residues involved in interactions or post-translational modification (PTM) sites within IDRs exhibit similar constraints as globular proteins [58].

Despite the extensive evidence, studies tend to focus on mutations within folded regions, sometimes neglecting or classifying mutations within IDRs as VUS. Considering these findings, it is crucial to thoroughly study the effects of variants in IDRs when prioritizing variants of RDs.

## Why explore the effect of variants in BCs?

Within the crowded cellular milieu, processes require precise spatiotemporal regulation and organization. Conventionally, this organization has been attributed to lipid membrane organelles. However, the emerging concept of biomolecular condensation demonstrates that fundamental cellular biochemistry extends beyond membrane barriers [29, 59]. These BCs selectively concentrate biomolecules in defined foci, leading to membraneless organelles (MLOs). The absence of membranes in these condensates facilitates rapid sensing and adaptation to environmental changes, allowing the exchange of their constituents with the surrounding cytoplasm or nucleoplasm without requiring specialized transporters [51]. The emergence of MLOs is mainly attributed to the liquid-liquid phase separation (LLPS) in biomolecules [60, 61] (see Fig. 1). Through this organizational mechanism, cells create unique environments by selecting specific components that regulate biomolecule availability, reducing noise in cellular computation and facilitating enhancing reaction rates [62–64]. This dynamic regionalization of components enables the precise orchestration of a myriad of cellular reactions and processes. BCs have thus emerged as primary organizers at different scales, and their role in both physiological and pathological processes has been fully demonstrated [32, 33, 60, 65–67].

BCs acting as hubs for signal modulation add another layer of complexity to phenotype determination [68, 69]. Therefore, understanding the interplay between variable expressivity and BCs is vital for deciphering pathological mechanisms and phenotypic heterogeneity in RDs. However, to the best of our knowledge, variant prioritization algorithms that address this have not yet been developed for RDs. This pending task can be facilitated by filtering variants as affecting scaffolds or clients [70, 71]. Scaffolds are biomolecules that self-associate through multivalent interactions, driving molecular condensation, while clients join this scaffolding, modulating the condensate’s composition and creating a liquid network of competitive interactions [72]. This process causes interacting components to segregate, leading to a condensed phase with higher biomolecule density, analogous to precipitation in saturated solutions. Specific proteins exhibit fundamental characteristics that promote condensate formation [51, 73–76].

IDPs and IDRs play a crucial role in cellular homeostasis through molecular condensation processes [29, 77]. In each protein, the primary structures and particular portions of linear sequences within it, as low complexity regions (LCRs) or short linear interacting motives (SLiMs), influence the formation and composition of the condensate [72, 73, 78]. These linear sequences encompass various interactions such as electrostatic interactions, π-π and cation-π contacts, hydrophobic interactions, and the valency and arrangement of LCRs [79–82] (see Fig. 1). However, the relevance of these sequences and the constraints governing their interactions are not yet fully understood [83–85]. Studies about the relationship between protein phase behavior and sequence modifications, such as deletions, truncations, or site-specific mutations, have revealed sequence-dependent characteristics that influence the phase separation of proteins [32, 33, 74, 86–88]. These include SLiMs and LCRs found within IDRs and modular interaction domains (MIDs) [32, 39]. MIDs are well-structured protein domains known for their essential functions in homo/heterotypic interactions among proteins, nucleic acids, or other molecules, i.e.: 14-3-3 domain, SH2 domain, Methyl-CpG DNA binding domain, etc. Unfortunately, the experimental challenges in studying IDRs limit our understanding of them. Recent discoveries highlight the pivotal roles of SLiMs, and LCRs in the formation of phase-separated condensates. SLiMs and LCRs, acting as mediators, play a crucial role in the selective partitioning and distinct composition of these condensates. By orchestrating such interactions, SLiMs and LCRs significantly contribute to shaping the architecture and functional diversity of cellular compartments, providing insights into the intricate mechanisms governing their formation and regulation [89]. Pappu and colleagues proposed the linkers-and-spacers model, which reduces this complexity to a pair of components: “linkers” as adhesive elements driving interactions, and “spacers” connecting stickers and influencing biomolecule-solvent interactions [78]. In IDRs, aromatic amino acids (Tyr or Phe) act as linkers, facilitating intra- and intermolecular contacts, while glycine and polar amino acids act as spacers without strong interaction patterns. Therefore, genetic variants on MIDs or IDRs can alter several aspects of BCs, including their formation, size, localization, material properties, and composition, consequently affecting the functional characteristics of BCs. This has been demonstrated in various pathologies, giving rise to the term “condensatopathies”: abnormal condensation leading to a specific disease phenotype [90] (see Fig. 1).

While only a subset of biomolecular components appears to be essential for maintaining condensate integrity [91–93], the potential number of these molecules within a condensate is vast, encompassing tens to hundreds of different biomolecules [94]. Thereby, condensates provide a platform for spatiotemporal regulation of cellular processes by self-organizing specific biomolecules and orchestrating their interactions [29].

Studying variant effects on condensate-promoting features like IDRs and their impact on BCs’ collective behavior can complement prioritization protocols, aiding in reclassifying VUS and enhancing diagnostics. Indeed, this approach may also provide valuable insights into phenotypic heterogeneity, missing heritability, and incomplete penetrance observed in RDs patients. We propose to analyze the effect of genetic variation in protein regions that promote condensation, such as IDRs, and their propensity to undergo phase separation due to the set of non-synonymous variants present in patients with RDs, using a single and multi-gene causation approach. These innovative strategies hold immense potential for identifying pathogenic variants, enhancing diagnostic capabilities for individuals affected by RDs, and elucidating their underlying molecular mechanisms, opening new avenues for therapeutic exploration.

To improve variant prioritization, we propose to study a new set of variables derived from in-silico predictors of disorder and condensation. We will assess the cumulative effect of patient-specific variants and their correlation with alterations in the composition of BCs, discriminating between linkers and spacers in scaffolds and clients. This comprehensive evaluation will elucidate the significance of individual components in disease manifestation and phenotypic diversity, providing deeper insights into the molecular underpinnings of disease and the relationship between genetic variations and phenotypes.

## The method

To gain a deeper understanding of the role of BCs in cellular organization and function, it is essential to compile an accurate annotation of the IDRs of the human proteome, a precise inventory of those proteins involved in BCs, and all the competitive interactions among them. In the field of IDRs, the accumulation of experimental evidence over two decades has robustly substantiated the notion that IDRs can be inferred from sequence features. This body of research has paved the way for the development of databases and [95] multiple IDR prediction methods, employing diverse principles and sophisticated computing techniques [96, 97]. These advancements have significantly enhanced our capacity to identify and characterize IDRs, thus deepening our understanding of their functional significance in protein structure and function. The same has happened in the field of BCs, where research in the past decade has advanced in the elucidation of the protein composition of different BCs and the characterization of their roles as scaffolds and clients [98–101], thus allowing cataloging them according to their propensity to undergo condensation in-vitro[102-106].

In the evaluation of the propensity of each protein to condensate, it is noteworthy that while scaffolds have been recognized as pivotal components [107], clients, which do not possess inherent phase separation capabilities, may influence the formation and regulation of BCs through their interactions with one or more drivers [72, 108]. However, it should be noted that many clients have not been thoroughly characterized or individually tested in vitro, and the existence of additional scaffold proteins cannot be ruled out [109]. This distinction between scaffolds and clients underscores the challenge of predicting whether a protein will localize into a BC and whether changes in the aminoacidic composition will affect the cellular self-organizing process. While all BCs proteins may share certain standard features, those distinguishing clients from scaffolds differ. Therefore, further research is needed to unravel the precise mechanisms underlying protein localization to MLOs and to gain a more comprehensive understanding of its various properties.

**Fig. 2:**
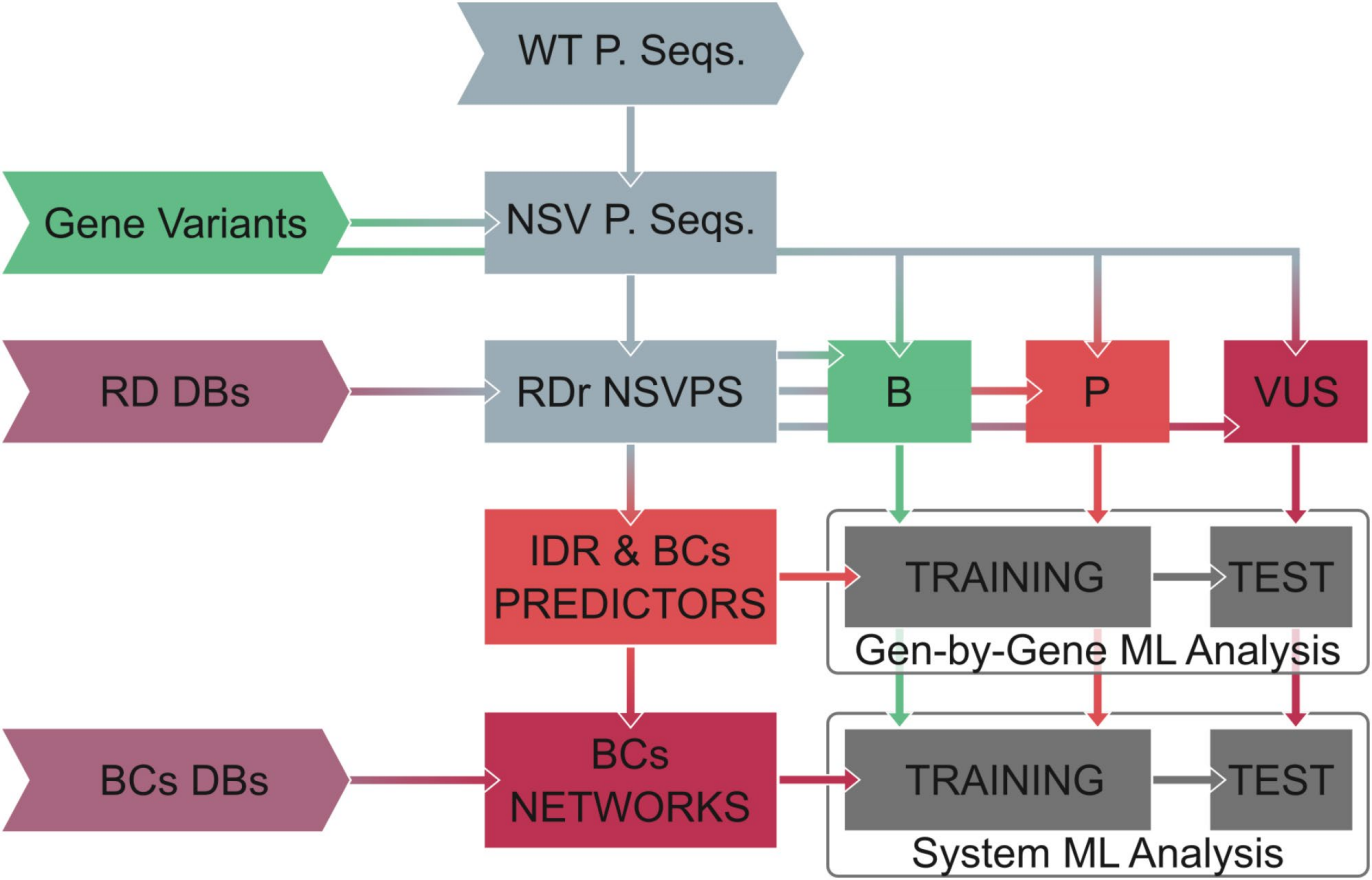
Schematic representation of the workflow. WT P. Seqs, Wild type protein sequences; NSV P. Seqs, Non-synonymous variant derived protein sequences; RDr NSVPS, Rare disease related non-synonymous variant derived protein sequences; B, Benign; P, Pathological; VUS, Variant of uncertain significance; RD DBs, Rare diseases databases; BCs DBs, Protein-protein interactions, and biomolecular condensates databases;

Despite these weaknesses, depicted advances have greatly enhanced our understanding of the various factors influencing molecular condensation. Based on this knowledge, new computational methods have been developed to accurately predict the propensity of proteins to remain disordered or undergo condensation [73, 84, 110–117].

Machine learning algorithms enable us to explore the categories of the American College of Medical Genetics and the Association for Molecular Pathology [118] and others for predicting variant pathogenicity. Algorithms are trained on pathogenic and population variant data using a wide range of features including evolutionary information (such as “conserved sites”), gene-level properties (e.g., “essentiality”), and specific amino acid substitutions in protein sequences [119–123]. While these methods aid in predicting causality and improving genetic diagnosis [124, 125], predictions generated are not always biologically interpretable, making it difficult to determine the reasons why a particular missense variant is predicted to have a high or low pathogenicity score.

In this perspective, we propose to apply machine learning algorithms to the information from multiple predictors, network analysis metrics, and database annotation to enhance the classification of VUS, leading to more informed clinical decisions (see Fig. 2).

For model training and validation, variants from Clinvar [126] are segregated into a training dataset (containing well-characterized pathological and benign variants) and a test dataset (comprising likely benign, likely pathogenic, and VUS variants). Data preprocessing ensures data quality and reliability. For feature selection, we adopt a multi-faceted approach, incorporating predictors of IDRs to identify disorder propensity, linear interacting peptides, arginine and tyrosine-enriched domains, and polyproline regions within the protein sequence. By incorporating IDR predictors, such as MobiDB-Lite [127], flDPnn [128], and Bio2Bite tools (Disomine [129], Dynamine [130], Efoldmine [131], and Agmata [131, 132]) we predict protein biophysical properties from their amino-acid sequences. This enables us to capture the propensity of specific regions within a protein to exhibit disorder, thereby highlighting the potential impact of a variant on the protein-disordered regions. Secondly, to evaluate the likelihood of a protein undergoing phase separation and forming BCs, we apply condensation propensity predictors. These predictors leverage sequence features associated with condensate formation, such as LCRs, prion-like domains, and specific amino acid compositions. By employing established algorithms like ParSe [110], LLPhyScore [115], MaGS [133], PScore [134], and PhasePre [114], we assess the condensation propensity of proteins and identify variants that may influence their ability to form or modulate BCs. Condensation propensity predictors are used to evaluate the global and local likelihood of a protein and its regions undergoing phase separation. Third, in addition to sequence-based features, we incorporate topological measures derived from a bipartite protein-protein interaction network labeled as scaffolds and clients. By analyzing network properties such as degree centrality, betweenness centrality, nestedness, fuzzy modular segregation, and assortativity, we gain insights into the relevance of proteins within cellular processes and a comprehensive understanding of the functional relevance of genetic variants in the protein-protein interactions network. This enables us to identify variants that may disrupt critical protein interactions and perturb cellular pathways, thus providing valuable insights into the clinical significance of the variants. Finally, database information such as Scaffold or client annotation or HPO and GO terms related to the proteins is added to improve interpretability.

By integrating these sets of features, we aim to capture a wide range of biological characteristics associated with genetic variants. The inclusion of IDR predictors allows us to identify regions of disorder within proteins, highlighting their functional relevance. Condensation propensity predictors provide insights into the potential for phase separation and condensate formation, elucidating the role of variants in cellular organization. Topological measures derived from protein-protein interaction networks further enhance our understanding of the functional impact of these variants in cellular processes.

The comprehensive set of features selected in our proposed method facilitates a multi-dimensional analysis of genetic variants, enabling us to redefine their clinical significance. By leveraging machine learning algorithms, including support vector machines, random forests, and neural networks, to develop robust classification models with these informative features, we aim to develop a robust model for variant classification and provide a more accurate assessment of variants classified as VUSes. We hope that our approach will improve clinical decision-making and increase our understanding of the functional implications of genetic variants in the context of genetic diseases. These models are trained using a carefully selected training dataset over the features previously described and learn to classify VUSes as either pathogenic or benign, thus improving variant classification.

To prioritize informative features and maximize interpretability in machine learning models, we propose utilizing various strategies that offer valuable insights into the variant classification process. These strategies include feature importance analysis, partial dependence plots, individual instance interpretation, rule-based models, and model-agnostic interpretability techniques [135, 136].

Feature importance analysis reduces model dimensionality and prioritizes informative features, enabling clinicians to focus on critical factors driving accurate classification decisions. Partial dependence plots and individual instance interpretation techniques, visualize the relationship between specific variables and model output, allowing a clear understanding of their impact on variant classification independently of other variables. Individual instance interpretation techniques may provide detailed explanations for classifying individual variants, highlighting key factors considered by the model in its prediction. Model-agnostic interpretability techniques, such as LIME and SHAP, offer post-hoc explanations for any black-box machine learning model. By perturbing input features and analyzing the model’s response, these techniques generate local explanations that help clinicians understand the factors influencing predictions for individual variants. Finally, to enhance interpretability, rule-based models such as decision trees or rule sets could be employed. These models map input features to predicted classes, providing transparent guidelines for clinical decision-making.

By incorporating these interpretability methods, clinicians will gain access to a comprehensive toolbox for understanding and interpreting predictions made by the proposed machine-learning models. These strategies provide transparent insights into the decision-making process, instill trust in the model’s predictions, and facilitate effective integration into clinical practice. Examining these explanations will enable clinicians to validate and interpret the model’s predictions on a case-by-case basis, enhancing the overall utility of the models in clinical decision-making.

## It won’t be an easy road

While the low frequency of each RD may seem insignificant for this type of study, in the US alone, approximately 30 million people are affected by RDs, impacting around 1 in 10 Americans [14]. Moreover, there are currently recognized between 5,000 and 10,000 RDs, depending on the source [137], providing a vast phenotypic landscape to explore the interdependence between variants and the unfolded phenotype.

Obviously, not all VUSes are linked to alterations in condensation processes. Exome sequencing covers less than 2% of the genome, allowing a diagnostic yield of around 30% [138] and leaving precise disease mechanisms largely unexplored [139]. Recent research has aimed at expanding the search space beyond coding regions to the immediate regulatory regions, revealing new pathogenic variants in a small fraction of cases [140]. Additionally, emerging reports suggest the involvement of distal enhancers and alterations in the three-dimensional (3D) genome structure in disease pathogenesis [141, 142]. Thus, the comprehensive exploration of the non-coding genome will provide valuable insights into the underlying mechanisms of genetic disorders and expand our understanding of the intricate regulatory networks that govern gene expression and cellular functions. In the context of the BCs these non-coding regions, whether expressed or not, have the potential to influence the cellular biomolecule composition. They can impact enhancers or promoter regions, alter the target selection of microRNAs, affect splicing variants, or influence transcript lifespan. Such changes can disrupt the critical balance of biomolecules involved in LLPS, thereby impacting condensation and the resulting biomolecular condensates’ composition. The reasons mentioned above further emphasize the necessity of adopting a systems biology approach. By integrating whole-genome sequencing, transcriptomic analysis, and computational models including biomolecular condensation propensity, competing RNA-RNA and RNA-protein interaction networks, and phenotypic enrichment, we can gain a comprehensive understanding of the underlying mechanisms of these diseases. This integrative approach could allow us to unravel the intricate interactions within biological systems and provide valuable insights into disease pathogenesis.

The fields of disorder and condensation prediction, as well as coarse-grained models of biomolecular self-organization, are rapidly evolving. However, it is important to note that predictors of disorder and condensation propensity, which rely on the primary sequence of proteins, have notable limitations [96, 117, 143]. The prediction of condensation propensity faces several challenges. For example, in the case of condensation propensity predictors, they commonly rely on a limited set of validated scaffolds for training algorithms, which greatly restricts their ability to accurately predict the condensation propensity of client proteins or other molecules also involved in the condensate. Additionally, our understanding of the underlying grammar of these processes is still very limited, and further experimental investigations are required to elucidate the logic behind condensation processes. Moreover, the role of post-translational modifications in triggering the condensation-decondensation process is well-known, but comprehensive data on these modifications for training machine learning algorithms are currently lacking. The prioritization of variants affecting linkers or spacers is a scientifically sound approach, provided the validity of the proposed model of linkers and spacers is acknowledged. It is crucial to recognize that models, albeit valuable tools, inherently reduce the complexity to achieve mathematical and computational tractability, potentially excluding critical information. Nevertheless, even with its limitations, employing a partial rule-based framework remains preferable to the absence of any guiding principles in variant prioritization.

Regarding disorder, the plasticity and interactivity of IDRs and their potential cellular function remain hard to predict [96, 127]. Understanding the grammar of IDRs is the first step on the path to deciphering these cellular self-organization processes. We lack experimental data about IDRs. However, given the experimental challenges associated with their study, multiple efforts are being made in the development of computational tools that enable us to delve deeper into this field, including the establishment of initiatives such as the Critical Assessment of Protein Intrinsic Disorder Prediction (CAPIDP) to set quality standards in the field. This highlights the continued interest in optimizing these predictors and in the need observed by the scientific community to access this valuable information for medical use.

## Concluding remarks

In recent years, the concepts of intrinsically disordered regions IDRs, BCs, and liquid-liquid phase separation LLPS determinants have significantly advanced the fields of molecular biology and genetics, providing novel insights into gene regulation, protein function, and the underlying biology of diseases. As these concepts have matured, the integration of this knowledge into the prioritization of genetic variants becomes increasingly compelling. By incorporating predictors of functional properties of IDRs and condensation propensity in variant prioritization, we can take advantage of the mounting evidence that highlights the crucial role of condensation processes in disease pathogenesis. This integration promises to improve diagnostic accuracy, unravel molecular mechanisms underlying rare diseases, and facilitate the discovery of novel therapeutic targets and pathways, enabling innovative interventions for complex disorders. Combining advanced computational models with precision medicine approaches opens new horizons for more effective treatments, driving forward rare disease research and enhancing patient outcomes.

As the scientific understanding of IDRs, BCs, and LLPS continues to advance, their integration into clinical practice becomes increasingly essential. A comprehensive grasp of the complexities of genetic variant pathogenicity, including the impact of condensation processes, is crucial for improving diagnostic accuracy and patient care. Embracing this evolving field and incorporating predictive tools into clinical workflows better equips us to address the challenges posed by extensive genetic diversity, variable expressivity, and incomplete penetrance associated with rare diseases. Ultimately, integrating these cutting-edge approaches into clinical settings will lead to a more personalized and precise approach to medicine, yielding improved patient outcomes and deeper insights into genetic diseases.

## Electronic supplementary material

Below is the link to the electronic supplementary material.

Supplementary Material 1

## Data Availability

https://osf.io/sntfu/?view_only=c8f0d139acf74bc4952c8aa9201279e0.

## Abbreviations

BCs: Biomolecular condensates
IDPs: Intrinsically disordered proteins
IDRs: Intrinsically disordered regions
LCRs: Low complexity regions
LLPS: Liquid-liquid phase separation
MIDs: Modular interaction domains
MLOs: Membraneless organelles
PTM: Post-translational modification
RDs: Rare diseases
SLiMs: Short linear interacting motives
VUS: Variants of uncertain significance
