# Supplementary material for: VUS next in rare diseases? Deciphering genetic determinants of biomolecular condensation
Source: Orphanet J Rare Dis. 2024 Sep 6;19:327. doi: 10.1186/s13023-024-03307-6 (PMC11380411; doi:10.1186/s13023-024-03307-6)
Supplement: Supplementary file 1 — Supplementary Material 1 [file 13023_2024_3307_MOESM1_ESM.pdf]

To Dr. Holm Graeßner,
Guest Editor
BMC Diagnosis of rare diseases strategies and structures

Re.: Manuscript of a *Position paper* to be submitted to the BMC Diagnosis of rare diseases strategies and structures

August 11st, 2023

Dear Dr. Graeßner,,

Please you can find enclosed the manuscript of a *Position paper* essay entitled ***VUS next in Rare Diseases? Deciphering genetic determinants of intracellular interactions***, to be submitted to the BMC Diagnosis of rare diseases strategies and structures.

The study of rare diseases (RDs) remains a persistent enigma in scientific exploration. The complex nature of these conditions, characterized by their scarcity and genetic intricacies, underscores the formidable journey ahead. At the bottom of this endeavor lies a mazelike puzzle that requires the integration of complementary paradigms and a reimagining of conventional diagnostic methodologies.

The canvas we explore is marked by genetic diversity, woven across nearly 8 billion individuals, each harboring an average of 5·106 genetic variants. Yet, this genetic mosaic merely hints at the layers of intricacy beneath. Deeper complexities arise from epistatic interactions orchestrating the modulation of phenotypic expression. These intricate challenges expose the incompleteness of traditional variant prioritization methods.

At the heart of this endeavor lies the accurate identification of causative variants—a task transcending clinical implications. Beyond guiding clinical decisions, it illuminates the path for future generations, shaping family planning and risk assessment. However, this journey is marred by delays, often spanning 4-5 years or, even decades. This is worsened by the increasing number of variants of uncertain significance.

Multiple pieces of evidence accumulated over the past decades show a complex choreography governing phenotype, one centered on the consequences of genetic variants shaping biomolecular condensates. These dynamic assemblages of gene products generate effects cascading through cellular behavior, offering a novel avenue for exploration.

Biomolecular condensates offer a key to decoding the intricate web of genetic diseases. Their implications extend beyond specific conditions, reaching areas like cancer and neurodegenerative disorders. These condensates possess the potential to fill the current gaps in genetics diagnostics and deeply understand the epistatic regulation of gene expression.

Despite their promise, the integration of these concepts into clinical practice remains nascent. We propose filling those gaps by the study of biomolecular condensation determinants as allies in deciphering the RD enigma.

Beyond clinical utility, these entities hold keys to unraveling complexities like variable expressivity and epistasis—cornerstones of rare genetic diseases, opening unexplored strategies of treatments. We propose a novel framework for the seamless integration of biomolecular condensates into routine clinical practice through the prioritization of variants by studying the effects of the non-synonymous variants of a patient in the processes of biomolecular condensation.

In summary, we are at a crossroads—a junction of tradition and innovation, complexity and clarity. Our journey is driven by an unwavering commitment to knowledge, grounded in the needs of patients and families.

For all the reasons presented above, we are convinced that contribute to deciphering the genetic determinants of intracellular interactions would be a very valuable contribution in the field. This is the aim of our *Perspective* manuscript, with the following table of contents:

1. The idea
2. Why explore the effects of variants in IDRs?
3. Why explore the effects of variants in IBCs?
4. The method
5. It won’t be an easy road

6. Concluding remarks

This manuscript also contains two figures. In the submitted figures, there is no image duplication, image manipulation, or visual plagiarism.

We are confident that our *position paper* would be of interest to the readers of the **BMC Diagnosis of rare diseases strategies and structures.**

All the co-authors of the manuscript agree with it submission as it is to the *BMC Diagnosis of rare diseases strategies and structures.* The authors declare that there is no conflict of interest.

Best regards,

Raúl Montañez
On behalf of all the co-authors

Suggested reviewers:

Lidia Castro Feijóo (lidia.castro.feijoo@sergas.es),

Begoña Izquieta (begona.ezquieta@salud.madrid.org),

Richard A. Young, (young@wi.mit.edu)

Lucas Pelkmans (lucas.pelkmans@imls.uzh.ch),

Denes Hnisz (hnisz@molgen.mpg.de),

Martin A. Mensah (martin-atta.mensah@charite.de),

Trever G. Bionova (trever.bionova@ucfs.edu),

Uros Midic (uros@temple.edu)
